# Evaluating the implementation of the standard treatment guidelines (STGs) and essential medicines list (EML) at a public South African tertiary institution and its associated primary health care (PHC) facilities

**DOI:** 10.1186/s40545-021-00390-z

**Published:** 2021-12-14

**Authors:** Tashni Govender, Fatima Suleman, Velisha Ann Perumal-Pillay

**Affiliations:** Discipline of Pharmaceutical Sciences, College of Health Sciences, University of KwaZulu-Natal, Private Bag X54001, Durban, 4000 South Africa

**Keywords:** Standard treatment guidelines, Essential medicines list, South Africa, Primary and tertiary health care, Rational use of medicines

## Abstract

**Background:**

The standard treatment guidelines (STGs) and essential medicines list (EML) were a policy option recommended in the National Drug Policy for South Africa in 1996 to address the irrational and bloated medicines procurement list. STGs/EML serve as a tool to promote cost-effective use of medicines; rational prescribing; and improve accessibility to medicines for all citizens. The purpose of this study was to evaluate the use and implementation of the STGs/EML by prescribers at a public tertiary institution and its associated Primary Health Care (PHC) facilities in the uMhlathuze subdistrict of KwaZulu Natal. The study aimed to provide feedback and to make recommendations to policy makers to improve the use and implementation of the STGs/EML and to inform National Health Insurance (NHI) policy development.

**Method:**

An observational quantitative descriptive research design was used. A retrospective audit of prescriptions was conducted, and questionnaires were utilized to collect data from prescribers and the facilities to evaluate the utilization of the STGs/EML and the rational use of medicines. All descriptive analyses were presented as counts with percentages, and Fisher’s exact test was used to compare results. The data was summarized, reduced, and analysed using SAS statistics software.

**Results:**

107 medical doctors (97%) responded to the questionnaire at hospital level and 98 nurses (98%) responded to the questionnaire at the PHC level. Results revealed that the majority of doctors, 94.4%, had access to the latest STGs/EML compared with only 41.8% of nurses. 70.3% and 78.3% of doctor’s and nurse’s prescriptions, respectively, adhered to the guidelines. 94.9% of nurses requested training on the use of STGs/EML as most of them had not received formal training on its use.

**Conclusion:**

This study showed suboptimal adherence to STGs/EML by all prescribers, in contrast to previous research amongst nurses when hardcopies were available. Training on the use of the STGs/EML is needed at PHC level and improved monitoring of STGs/EML compliance is necessary. This study may be replicated for a wider population to paint a national picture and to periodically assess the extent of the implementation of the STGs/EML.

## Background

To meet the basic constitutional human right to healthcare, the new democratic South African government commissioned a committee to look specifically at medicine issues, which gave rise to the National Drug Policy (NDP) of 1996. The NDP sought to address deficiencies such as the irrational use of medicines, inaccessibility to medicines and cost-ineffective treatment, and inefficient procurement and logistic practices to “ensure an adequate and reliable supply of safe, cost-effective drugs of acceptable quality to all citizens of South Africa and the rational use of drugs by prescribers, dispensers and consumers”. The NDP allowed for the provision of the Essential Medicines Lists (EML) guided by the Standard Treatment Guidelines (STGs) through the National Essential Drugs Programme [1]. The NDoH provisioned the development and maintenance of the STGs/EML through the National Essential Medicines List Committee appointed by the Minister of Health, which is supported by the Affordable Medicines Directorate (AMD). The AMD is a directorate with the NDoH which is responsible for the development of systems to ensure access to essential pharmaceutical services and provides provinces with legislation and policies (regulatory frameworks) to monitor access to medicines [2].

STGs are intended to provide therapeutic guidance that is necessary to treat the prevalent health conditions of a country [3]. The EML is derived from the STGs and consists of a limited range of medicines that are intended to be available at all times in sufficient quantities and are chosen with due regard to efficacy, safety, and affordability [4]. The South African STGs/EML provide background information on each medical condition and recommended treatment regimens linked to the medicines listed in the EML, as well as non-pharmaceutical interventions, per level of care [2].

The STGs are evidence-based guidelines, with an emphasis on efficacy, safety and cost-effectiveness to treat the priority health conditions of a country’s population. Using rational medicine selection, the EML streamlines the number of medicines required to treat the prevalent health conditions according to guidelines set by the STGs. This results in a limited number of medicines that need to be procured by the country’s public health sector which allows for the efficient allocation of public financial resources; and also streamlines the supply chain process through efficient drug procurement and inventory control; as well as creates competition amongst suppliers translating to lower tender prices leading to greater accessibility to essential medicines and improved health outcomes [3, 4]. STGs/EML utilization in SA is a priority in the public sector but has also been encouraged in the private sector as they serve to standardize patient care and provide a basis to monitor and assess the quality of care; and aids medicine procurement and inventory control processes [5, 6]. Non-compliance to the STGs/EML results in increased opportunity costs and poor health outcomes such as an increased risk of adverse drug reactions, treatment failure, polypharmacy and antimicrobial resistance, and ultimately results in the inefficient use of resources which directly impacts the availability of medicines [2].

Essential medicines, as a concept, is dynamic and requires the process of selecting medicines to continually review new therapeutic choices according to the healthcare needs and changing disease patterns of the population while ensuring medicine quality, safety and affordability [5]. The EML and the STGs allow provincial formularies to be drawn up. The South African public healthcare sector remains resource-constrained, while the healthcare needs of the population continue to grow. New medicines and treatment regimens continually enter the market with the promise of improved health outcomes, but at a high-cost burden [2]. This requires the revision and review of STGs/EML to ensure that the best available treatments are accessible and financially sustainable.

State healthcare establishments are categorized according to the type of healthcare services rendered in terms of Section 35 of the National Health Act, 2003 (Act No, 61 of 2003). Primary level consists of Primary Health Care (PHC) clinics and community health centres; secondary level consists of district and regional hospitals; and tertiary/quaternary level consists of tertiary and central hospitals [2, 7]. The first STGs/EML were published in 1996 for the primary healthcare level, which was subsequently followed by secondary (hospital) healthcare STGs/EML, and only a recommended EML for tertiary and quaternary healthcare [5, 8]. To strengthen the public healthcare system and provide widespread access to healthcare, the NDoH introduced a policy in 1996, called “Restructuring the National Health System for Universal Primary Health Care” [9]. This policy provided the framework by which PHC services would be rendered and specified the role of a designated group of nurses who served as the backbone of PHC services at public facilities. This policy, along with the country’s nursing regulatory body, the South African Nursing Council through the Nursing Act, No. 33 of 2005 as amended, allowed for this specialized group of professional nurses to function independently to diagnose and treat patients at public PHC facilities [9, 10].

Previously, new editions of the STGs/EML were published as hardcopies and disseminated across the country. This approach was met with challenges as the review process was ongoing, resulting in out-dated versions of the guidelines. Thus, the NDoH introduced the first free mobile application for the STGs/EML in 2015 known as the EML Clinical Guide, to improve accessibility to the guidelines and ensure that the latest information was readily available [11]. Since the introduction of the mobile application, there have been no studies done to evaluate the impact on accessibility to the guidelines. There are limited studies conducted in South Africa to evaluate the improvement of medicine use and accessibility through policies, such as the STGs/EML. Particularly, in developing countries, the implementation and evaluation of STGs/EML has not been simple as these countries lack the infrastructure and resources to develop a comprehensive evaluation and monitoring system to evaluate these policies [12].

A global study that analysed the data of 23 countries from the World Health Organization (WHO)/Health Action International database in 2014 looked at the implementation of STGs/EML and found that essential medicines were more available than non-essential medicines in all health care institutions (both public and private) due to essential medicines being prioritized through policy interventions; however, the availability of essential medicines is not yet satisfactory [13]. The average availability of essential medicines in developing countries (SA included) was found to be 34% in public facilities compared to 63.2% in the private sector [14].

In SA, poor healthcare infrastructure and high medicine prices were identified as the factors to most adversely affect the implementation of the EML. The lack of resources has resulted in over-strained public health facilities, with limited hospital capacity, personnel shortages, and poor procurement and supply systems, which have further impacted EML implementation in the country [14].

Improved health outcomes and increased life expectancy are considered a social investment; thus, the State recognizes that the implementation of universal health coverage (UHC) is necessary for poverty reduction, sustained economic growth and socio-economic development [15]. To transform an unequal health care system, South Africa is in the process of implementing UHC, through National Health Insurance (NHI), which is a single financing system intended to provide all South Africans with essential health care [16].

NHI will provide medicines on the National Reimbursement Medicines List to patients who are treated in accordance with the STGs; therefore, strategic purchasing and procurement systems must ensure that access to healthcare is improved while delivering services cost-effectively and based on scientific evidence by utilizing clinical guidelines (viz. STGs/EML) to guide treatment strategies and prevent excessive use of healthcare services to ensure the sustainability and affordability of the fund [16], hence the need to evaluate the use and implementation of the current STGs/EML in public health institutions and facilities in South Africa to inform such policy changes. There is a need for research of this nature to assess the prescribing of essential medicines and the availability of essential medicines for priority diseases [17]. Therefore, this study aimed to evaluate the implementation and utilization of the STGs/EML by healthcare workers, determine the extent of training and knowledge of prescribers on the use and implementation of these guidelines, and review the rational use of medicines in accordance with the guidelines at service delivery level.

## Methodology

An observational quantitative descriptive research design was used. This allowed the participants to reflect on how the STGs/EML were used within the public healthcare sector at PHC facilities and a tertiary hospital and provided data to make recommendations in practice [18]. To assess the key areas and achieve the objectives of this study three tools were developed and utilized. In part one of the study, a prescription audit was conducted, while in part two, a questionnaire was utilized to collect data from professional nurses and medical doctors at the institution and facilities to evaluate the utilization of the STGs/EML and the rational use of medicines, as well as to determine the extent of training and knowledge of the clinicians on the use of the guidelines. Finally, a tracer medicines list tool was utilized to determine the availability of medicines at each facility. Questionnaires were self-administered (and were in English), and the prescription audit was conducted by the researcher. Participation in the study was voluntary after informed consent was sought and anonymity of participants was maintained as no identifying data was collected. Data was collected from 7th January 2020 to 6th February 2020 during which all facilities were visited by the researcher upon receipt of ethics approval.

### Study site

The study was conducted within the subdistrict of uMhlathuze in the King Cetswhayo district of the province of KwaZulu-Natal at a tertiary hospital and 13 of its associated PHC clinics. The tertiary hospital also functions as a level 1 and level 2 hospital that caters to referrals from 18 hospitals in the northern KwaZulu Natal region [19].

### Sample recruitment

Questionnaires were distributed and prescribers’ prescription audits were conducted in 13 PHC clinics and 1 tertiary public hospital. Professional nurses at the primary care level were selected to be surveyed, as they function as prescribers in frontline care at public primary healthcare facilities in SA, as well as medical doctors at the hospital [9]. Medical doctors were approached, in person, from varying disciplines and levels (from interns to heads of clinical units). All participants were approached by the researcher on-site and were provided with an information sheet, which explained what the study was about and why they were being asked to participate, as well as an informed consent form which was signed by the participant before commencement. The participants were informed that they could withdraw from the study at any point.

### Inclusion and exclusion criteria for the questionnaire part of the study

Inclusion criteria:

- Professional nurses at PHC level who were qualified to prescribe.
- Doctors at hospital level.

Exclusion criteria:

- Feedback from participants who withdrew consent.
- Nurses who were not qualified to prescribe, such as enrolled nurses, student nurses and nurses based at hospitals.

### Inclusion and exclusion criteria for the prescription audit part of the study

Inclusion criteria:

- Patient records that contained a prescription with prescribed medicines within the previous 3 months of the researcher’s visit to the facility were assessed. The most recent prescription was evaluated to ensure that the prescribed treatment was in accordance with the latest guidelines.
- All prescriptions, irrespective of patient demographics or prescribed treatment, were included to allow for a full evaluation of the STGs/EML.

Exclusion criteria:

- Doctors from the mother hospital consult at each PHC facility once a week, thus doctor’s prescriptions at PHC level were excluded from the PHC prescription sample.
- Prescribers are required to indicate their qualification when signing a prescription; where no qualification was indicated the prescription was not included in the sample.
- Patient records that did not contain a prescription with prescribed medicines within the previous 3 months of the researcher’s visit to the facility.

### Sampling

All sample size calculations were performed by a statistician and allowed for a 5% margin of error with a 95% level of confidence. The formula used to calculate all sample sizes was

$$n = {{N*X} \mathord{\left/ {\vphantom {{N*X} {\left( {X + N - 1} \right),}}} \right. \kern-\nulldelimiterspace} {\left( {X + N - 1} \right),}}$$

where

$$X = {{Za} \mathord{\left/ {\vphantom {{Za} {22}}} \right. \kern-\nulldelimiterspace} {22}}*{{p*\left( {1 - p} \right)} \mathord{\left/ {\vphantom {{p*\left( {1 - p} \right)} {E2}}} \right. \kern-\nulldelimiterspace} {E2}}$$

and *Za*/2 is the critical value of the normal distribution at *α*/2, *E* is the margin of error, *p* is the sample proportion, and *N* is the population size. A finite population correction was applied to the sample size formula.

### Prescription audit sample size

The number of prescriptions recorded at the hospital per month was 6635, thus the minimum sample size of medical doctors’ prescriptions to be reviewed was calculated to be 37. The collective number of prescriptions recorded at the 13 PHC clinics per month was 46,966, thus the minimum sample size of nurses’ prescriptions to be reviewed was calculated to be 263. This sample size was divided proportionally to show the number of prescriptions to be reviewed per clinic (Table 1).

**Table 1:** Proportional questionnaire and prescription audit sample sizes at PHC facilities

Clinic	No. of prescribing nurses	No. of nurses to be surveyed for questionnaire	No. of prescriptions per month	No. of prescriptions to be reviewed for prescription audit
Facility 1	18	13	7240	41
Facility 2	16	12	2837	16
Facility 3	16	12	6239	35
Facility 4	4	3	1460	8
Facility 5	4	3	2400	13
Facility 6	17	12	4814	27
Facility 7	16	12	7124	40
Facility 8	5	4	1659	9
Facility 9	4	3	1005	6
Facility 10	9	6	1787	10
Facility 11	8	6	2185	12
Facility 12	11	8	6801	38
Facility 13	6	4	1415	8
Total	134	98	46,966	263

### Questionnaire sample size

The total number of medical doctors at the hospital was 147, thus 107 was the minimum sample size recommended to be surveyed. The total population of prescribing nurses was 134 at the 13 PHC facilities, thus 98 nurses was the minimum sample size recommended to be surveyed. This sample size was divided proportionally to show the number of nurses to be surveyed per clinic in the table (Table 1).

### Data collection tools and data collection process

#### Tool development and data collection process

The study evaluated the use and implementation of the STGs/EML by assessing the following key areas:

- Accessibility to STGs/EML:
  Determination of the accessibility of the STGs/EML to prescribers gave insight into how the guidelines have been used and implemented, and whether the STGs/EML policy had resulted in greater accessibility of the guidelines at the grassroots level.
- Frequency of use of the STGs/EML:
  Reporting on the frequency of use of the STGs/EML by prescribers gave insight into how the guidelines have been used and implemented, and whether the introduction of the STGs/EML had been a necessary and useful tool at the grassroots level.
- Availability of medicines:
  The STGs/EML were introduced to improve the accessibility of essential medicines to patients. Determination of the availability of essential medicines gave insight into how the guidelines have been utilized and implemented, and whether the STGs/EML policy had resulted in greater accessibility of medicines to, and improved treatment of patients.
- Rational use of medicines and adherence to the STGs/EML:
  Clinicians’ diagnosis/es were compared to the medicines prescribed in accordance with the STGs/EML which provided insight into whether the STGs/EML policy had ensured that the healthcare needs of patients were appropriately and safely met.
- Training and knowledge on use of the STGs/EML:
  The extent of training and knowledge on the use of the STGs/EML of prescribers by assessing prescribers’ responses on whether they had received formal training on how to use the STGs/EML, whether they were confident in their knowledge on how to use the STGs/EML and whether they wished to receive training on how to use the STGs/EML was assessed. This gave insight into how well the STGs/EML were understood at grassroots level and whether interventions were required to improve use of the guidelines.

The development of the three data collection tools was adapted from the first impact study conducted in South Africa by the South African Drug Action Programme in 1999 after the introduction of the first EML [20]. Data was collected using the three tools as follows:

1. The prescription audit tool was developed to determine the rational use of medicines at the facilities in line with STGs/EML recommendations to evaluate adherence to the guidelines by surveying prescriptions retrospectively. The prescription audit tool was designed to allow the researcher to record the diagnosis and treatment prescribed on the prescriptions sampled and then record whether the prescribed medicines were available on the latest EML and if the treatment prescribed complied with STG recommendations. The prescription audit was piloted by the researcher at a facility to determine the validity and ease of use of the tool, after which no changes to the prescription audit tool were found to be necessary. The prescription audit was conducted by the researcher on-site by observation to collect the data. Upon visiting each facility, the researcher evaluated prescriptions in the patient records, as per sampling protocol to document treatment compliance and the availability of the prescribed medicine. Systematic sampling was employed, i.e., every third prescription was analysed to increase representativeness and was easy to implement [21]. The prescribed medicines were reviewed against the STGs/EML to determine the appropriateness of treatment and the adherence of prescribed medicines to the STGs/EML. Prescriptions were considered as non-adherent to the STGs/EML if the incorrect medicines or regimens were prescribed in relation to the diagnosis according to the guidelines, the medicines were redundant, a medicine was contraindicated, the incorrect dose, medicine or duration of treatment was prescribed, polypharmacy was present, and if no diagnosis was recorded as it is legally required and necessary to determine rational medicine prescribing [22].
2. Questionnaire for survey: A self-administered questionnaire was developed and utilized which sought personal input from the participants on their experience with the use of the STGs/EML. The questions were closed-ended to quantify the responses. The questions sought to determine whether the prescribers had access to the latest STGs/EML (viz. the mobile application, EML Clinical Guidelines, and soft copies available from the NDoH website); had received training on how to use the STGs/EML; whether they would like to receive training on how to use the STGs/EML; and how often they had used the guidelines. For the purpose of this study the STGs/EML were considered accessible if available to the prescriber when required. In this study the STGs/EML were deemed accessible if the prescriber had access to the mobile app which contained the most up-to-date guidelines as hardcopies of the STGs/EML were no longer available and out-dated. The frequency of use of the STGs/EML was self-reported by prescribers by stating how often they used the STGs/EML by choosing from the options presented in the questionnaire. The questionnaire and a consent form were handed to the participants by the researcher for self-completion. The questionnaire was piloted with 10 prescribers from two facilities (both doctors and nurses) to ensure comprehension of the questions, ease of use and to rule out ambiguity. It was found that no changes to the questionnaire were required after being piloted.
3. Tracer medicines list tool: The National Core Standards (NCS) for health establishments in South Africa was developed as a benchmark to measure performance and maintain standards of quality health care and service delivery in all health facilities [23]. The tracer list tool used in this study to measure the number of key medicines available was based on a condensed list of medicines from the EML that were chosen as part of the NCS audit tool to monitor the availability of those essential medicines to evaluate the implementation of the STGs/EML. The tool listed 33 medicines, in the form of a checklist, and allowed the researcher to record whether each medicine on the tracer medicines list was present or absent in the pharmaceutical storeroom of each facility. This allowed the researcher to assess whether the essential medicines within the basket were accessible to patients if it was prescribed, and whether medicines prescribed by the clinician were listed in the STGs/EML to determine whether the pharmaceutical health needs of the patients were met. The designation of a medicine on an EML is dependent on its dosage form and indication, not its pack size. A medicine on the checklist was considered to be available if the listed item was present in the stated dosage form and strength (to account for paediatric and adult dosing requirements), regardless of the quantity present, within the pharmaceutical storeroom of the facility. The tool was piloted at one facility to determine the validity and ease of use of the tool, after which no changes to the tracer list tool were found to be necessary.

A data collection summary tool was developed and utilized to collate data collected using the prescription audit tool, questionnaire and tracer medicines list tool to measure the impact of the STGs/EML on medicine utilization and rational prescribing which allowed for easy analysis.

### Data analysis

All descriptive analyses which were stratified by designation (doctor/nurse) and site (clinic/hospital) were presented as counts with percentages. The Fisher’s exact test was used to compare the use and implementation of the STGs/EML, rational use of medicines at the institution in line with STGs/EML recommendations, the extent of training and knowledge on the use and implementation of the STGs/EML by healthcare professionals at the institution and facilities. This allowed the data to be summarized meaningfully and show patterns that emerged [24]. The data was reduced and analysed with the help of a statistician using SAS statistics software version 9.4 (SAS Institute INC., Cary). A two-tailed value of *p* < 0.05 was considered to indicate statistical significance.

### Ethical considerations

This study was approved by UKZN Biomedical Research Ethics Committee (approval number BE541/17) and the KwaZulu-Natal Department of Health National Health Research Database (Ref: KZ_201805_014). Participants were provided with an information sheet which explained what the study was about and why they were asked to participate—if the participant agreed to participate in the study they were required to sign and date a declaration of informed consent form.

No identifying data from the participants or patients were collected and used in this study, thus participation remained anonymous. Participation was completely voluntary, and participants were made aware that they could withdraw from the study at any stage without consequence. There were no incentives or direct benefits offered to the participants to participate.

## Results

### Demographics of the sample

A total of 107 medical doctors (97%) responded to the questionnaire at hospital level and a total of 98 nurses (98%) responded to the questionnaire at the PHC level (Table 2).

**Table 2:** Demographic information of participants

Demographic parameter	Doctors (*n* = 107)		Nurses (*n* = 98)	
	Frequency	Percentage (%)	Frequency	Percentage (%)
Age (years)				
20–29	17	1.89	21	21.43
30–39	39	36.45	41	41.84
40–49	38	35.51	27	27.55
50+	13	12.15	9	9.18
Average age	39		37	
Gender				
Male	66	61.68	9	9.18
Female	41	38.32	89	90.82
Experience (years)				
1–4	22	20.56	25	25.51
5–9	32	29.91	32	32.65
10–14	34	31.77	29	29.59
15+	19	17.76	12	12.25
Average years of experience	9		8	
Qualification/level				
Intern	18	16.82		
Community service	11	10.28		
Medical officer	69	64.49		
Specialist	9	8.41		
Professional nurse			98	100

### Analytical information

Table 3 (below) contains the combined results from the prescription audit (part one) and the questionnaire (part two) as the outcomes are presented according to the objectives of the study. Results for each respective part of the study are indicated by a key within the table.

**Table 3:** Prescription audit (part one) and questionnaire (part two) results assessing the implementation of the STGs/EML

	Doctor (hospital)			Nurse (PHC)				*p* value
	*n*	Frequency	%	*n*	Frequency		%	
**Use and implementation of the STGs/EML**								
Prescribers with access to the latest STGs/EML (mobile application)^#^	107^β^	101	94.4	98^β^	41		41.8	< 0.001
Prescribed medicines that required buy-outs for non-EML item*	37^α^	2	5.4	263^α^	0		0	0.015
Frequency of use of the STGs/EML by prescribers^#^								
Often	107^β^	17	15.9	98^β^	77		78.6	< 0.001
Sometimes	107^β^	85	79.4	98^β^	21		21.4	
Rarely	107^β^	5	4.7	98^β^	0		0	
Never	107^β^	0	0	98^β^	0		0	
**Rational use of medicines and compliance to STGs/EML**								
Prescriptions with diagnosis recorded*	37^α^	26	70.3	263^α^	206	78.3		
Prescriptions with diagnosis listed in the STGs/EML*	37^α^	23	62.2	263^α^	201	76.4		
Prescription adhered to STGs/EML*	37^α^	20	54.1	263^α^	157	59.7		
**Extent of training and knowledge of STGs/EML**								
Prescribers trained on the use of the STGs/EML^#^	107 ^β^	11	10.3	98^β^	17	17.3		0.158
Prescribers knowledgeable on the use of the STGs/EML^#^	107^β^	100	93.5	98^β^	33	33.7		< 0.001
Prescribers who wish to receive training on the use of the STGs/EML^#^	107^β^	39	36.4	98^β^	93	94.9		< 0.001

*Prescription audit (part one) result

^#^Questionnaire (part two) result

^$^Number of prescribers

^α^Number of prescriptions

Within each category of prescriber, viz. doctors and nurses, the number of prescriptions and the number of prescribers analyzed are indicated by a key within the table.

Within the prescriber category of doctors, the number of prescriber questionnaires analyzed was *n* = 107 and the number of prescriptions audited was *n* = 37. Within the prescriber category of nurses, the number of prescriber questionnaires analyzed was *n* = 98 and the number of prescriptions audited was *n* = 263.

Most doctors had access to the latest STGs/EML (94.4%; *n* = 101), which is now available as a mobile phone app, but only (41.8%; *n* = 41) of nurses surveyed had access to the latest guidelines. The majority of doctors stated that they sometimes used the STGs/EML (79.4%; *n* = 85) vs (21.4%; *n* = 21) of nurses, whereas the majority of nurses used the STGs/EML often (78.6%; *n* = 77) vs (15.9%; *n* = 17) of doctors. (4.7%; *n* = 5) of doctors used the STGs/EML rarely. None of the doctors or nurses stated never using the STGs/EML.

Of the prescriptions surveyed, two non-EML items (5.4%) were prescribed in the hospital level prescriptions which required a buy-out, where a medicine that is not on the EML is required for a patient. None of the PHC level prescriptions required a buy-out.

Only (10.3%; *n* = 11) of doctors and (17.3%; *n* = 17) of nurses stated that they had received training on the use of the STGs/EML, yet (93.5%; *n* = 100) of doctors considered themselves knowledgeable on the use of STGs/EML, whereas only (33.7%; *n* = 33) of nurses responded positively. Most nurses wish to receive training on the use of the STGs/EML (94.9%; *n* = 93), but only (36.4%; *n* = 39) of doctors responded similarly.

Doctors and nurses scored similarly for indicating the diagnoses on the prescriptions (70.3%; *n* = 26) and (78.3%; 206), respectively. Of those prescriptions, (62.2%; *n* = 23) of doctors’ diagnoses were listed in the STGs and (76.4%; *n* = 201) of the nurses’ diagnoses were found in the STGs. (54.1%; *n* = 20) of doctors’ and (59.7%; *n* = 157) of nurses’ prescriptions adhered to the STGs/EML. 62.2% of the diagnoses recorded by doctors and 76.4% by nurses were listed in the STGs. Due to the lack of recorded diagnoses on prescriptions, the findings for the lack of adherence to the STGs/EML may not be purely due to irrational prescribing and may also affect the number of diagnoses found listed in the STGs/EML. All facilities had between (93.9%;* n* = 31) to (100%;* n* = 33) availability of the basket of tracer medicines. 10 of the 14 facilities surveyed had (100%;* n* = 33) of the tracer medicine available (Table 4).

**Table 4:** Tracer medicines available per facility

Facility	Frequency	% Medicines available
Facility_1	33	100
Facility_2	32	97
Facility_3	33	100
Facility_4	32	97
Facility_5	33	100
Facility_6	33	100
Facility_7	33	100
Facility_8	33	100
Facility_9	31	93.9
Facility_10	33	100
Facility_11	33	100
Facility_12	33	100
Facility_13	32	97
Facility_14	33	100

## Discussion

### Main findings

The STGs/EML were much more accessible amongst doctors (who are based at the hospital in a more urban area) than nurses (who are based at clinics, often, in rural settings). The latest STGs/EML are now only available as a soft-copy on the NDoH website and as a mobile app. Prior to the mobile application being made available, after extensive peer-review revision, new editions of the STGs/EML were published every 3 years as hardcopies and distributed to public healthcare workers across the country. This approach resulted in many flaws as the review process is ongoing, resulting in frequently updated guidelines, and consequently out-dated versions of the guidelines were being utilised at institutions. To combat this, the NDoH introduced the first free mobile application for the PHC STGs/EML in late 2015 and subsequently included the hospital level STGs/EML in 2017, known collectively as the EML Clinical Guide. This was intended to improve accessibility to the guidelines and ensure that the latest information was readily available as the ownership of mobile phones is widespread and continually growing in SA [11]. The majority of doctors had access to the STGs/EML through the mobile app, compared to very few nurses. Challenges in accessing the latest STGs/EML, particularly amongst nurses, may be the lack of technological know–how and inability to navigate the internet and the app, personal data costs and the lack of access to a smartphone or computer. PHC facilities are also often located in distant and under-serviced areas, where internet access and mobile phone reception is poor. Although not formally surveyed, most respondents at PHC level revealed that they were not aware that the latest STGs/EML were available as a mobile app and that the hardcopies were out-of-date as the STGs/EML have been updated electronically.

Overall compliance to STGs/EML was found to be suboptimal, although the lack of compliance to STGs/EML was largely due to the lack of recording of diagnoses for both categories of clinicians. The lack of diagnoses was considered as non-compliant to the guidelines for the purpose of this study as the stating of diagnoses is a medico-legal requirement which may have resulted in an over or under-estimation of actual irrational medicine prescribing [22]. Nurses scored marginally better than doctors in both adherence to guidelines and the stating of diagnoses. The lack of adherence to guidelines can be affected by the complexity of both the diagnosis and the guidelines itself—where nurses see simpler, primary health care conditions for which treatment guidelines are straight-forward and the more complex health conditions (which may include multiple diagnoses) are referred to hospital to be seen by doctors and may have more complex diagnoses [25]. It is unknown whether the lack of recorded diagnoses was due to uncertainty of the diagnosis or simply the lack of recording it.

Although very few prescribers from both categories had received training on the use of the STGs/EML, the majority of doctors considered themselves knowledgeable on the use of the STGs/EML and did not wish to receive training on the use of the STGs/EML—a finding that was in stark contrast to the findings amongst nurses who wished to receive training. This result may indicate the ease of use of the mobile app by those familiar with and adept at using technology.

The majority of doctors stated that they sometimes use the STGs/EML, whereas the majority of nurses stated that they often used the guidelines, indicating that the STGs/EML are integral to developing treatment plans for patients. Prescribers are reliant upon those guidelines as all prescribers make use of the guidelines, yet less than half of all nurses had access to the latest STGs/EML which may result in suboptimal treatment plans for patients and impact the adherence to guidelines. Only a small number of doctors reported using the STGs/EML rarely which may be related to their field and the complexity of patients' conditions as well as the prescriber's experience.

The STGs/EML were introduced to improve rational medicine prescribing and improve availability and accessibility to medicines by streamlining the number of key medicines required to treat the health needs of a country, thereby improving the supply chain management and drug procurement systems [5]. The findings in this study indicate that EML do not guarantee absolute availability as stock-outs do occasionally occur, but the introduction of the STGs/EML has had a positive effect on the availability of essential medicines as most facilities had all the key basket medicines available upon inspection with only 4 facilities scoring between 97 and 93.9%, which well exceeded the WHO target of 80% [26]. This may be due to the surveillance systems, which utilize software for the weekly reporting of stock levels by clinics and hospitals, introduced in 2016 by the NDoH to proactively monitor stock levels to prevent stock-outs [27, 28].

The use of medicines not listed in the EML, termed non-EML medicines, is possible at state facilities on an individual patient basis. The principle of making non-EML medicines available in state facilities ensures equitable access to medicines as some health conditions may not respond to medicines and guidelines as per the STGs/EML. Non-EML medicines which do not have an attached STG must have a treatment protocol which follows the same format as the national STGs/EML to guide the use of the medicine and must be prepared by the relevant Pharmaceutical Therapeutics Committee. A prescription for a non-EML medicine must be accompanied by an approved application form containing a motivation for use submitted by the treating clinician for approval [2].

Two non-EML medicines were prescribed which required a buy-out utilizing applications for the use of non-EML medicines on a “named patient” basis, which indicated that the majority of the prevalent health conditions of the population were provisioned for by the treatment guidelines but that some exceptions to care exist in rare instances. STGs/EML provide recommendations based on the best clinical evidence available but are there to serve as a guide and cannot replace sound clinical judgement [25].

### Comparisons with other studies

The inaccessibility to treatment guidelines, particularly at the PHC level, is a common problem in Africa. Nine years after the introduction of STGs in Nigeria, a study found that 70% of clinicians did not have access to guidelines [29], these findings were echoed by a study in Botswana and Sudan, despite the guidelines being freely available online [30, 31]. Four of the earliest surveys in SA to evaluate the accessibility of STGs/EML amongst healthcare workers were conducted between 1996 and 2005 [32–35]. The number of prescribers with access to the STGs/EML rose considerably from 59% in 1998 to 97% in 2003 [32–34]. The 2005 survey considered the STGs/EML accessible if one guideline document was present within a public hospital and reported 100% accessibility [35]. Sooruth et al. (2015) [36] conducted a study in 2013 in the uMgungundlovu district of KwaZulu Natal, when hardcopies of the STGs/EML were in circulation in SA, and found that 100% of the nurses sampled had access to the STGs/EML. This is in stark contrast to the findings of this study amongst PHC nurses, who considerably lacked access to the online guidelines. After the STGs/EML were updated in SA in 2014, roadshows on the development processes of the STGs/EML were conducted in 2015 in three provinces, where it was found that over half the participants were not aware of the updated guidelines. Towards the latter part of the roadshow in 2016, after the introduction of the STGs/EML Clinical mobile app in late 2015, another survey revealed that just under 60% of participants at the roadshow surveyed were aware of the PHC STGs/EML mobile app and under 40% had used the app [11]. The lack of accessibility to the STGs/EML amongst PHC clinicians in this study suggests that the move to online STGs/EML may not have had the desired outcome of improved accessibility to the most up-to-date guidelines amongst all categories of healthcare workers, or that the roadshows were not successful and other educational interventions are required.

The lack of compliance to the STGs/EML of both doctors’ and nurses’ prescriptions can be largely attributed to the lack of recording of diagnoses in patient records. Likewise, Gasson et al. (2018) conducted a study in Cape Town, South Africa, which found that 30.5% of prescriptions had an unknown diagnosis during a study into antibiotic prescribing adherence to treatment guidelines and that doctors were less likely to record a diagnosis than nurses [37], in line with the findings of this study.

This study’s suboptimal guideline compliance findings were consistent with the many adherence studies conducted internationally in several counties, viz. Nigeria, Sierra Leone, United States of America, Switzerland and Palestine [38–42]. An Italian review that explored reasons for non-compliance to treatment guidelines found that more than 10% of prescribers ignored available treatment guidelines, prescribers were not fully aware of the contents of treatment guidelines and 10% of prescribers disagreed with the contents for various reasons, including the lack of credibility of the information provided, and the loss of autonomy in choosing treatment options [43].

There have also been numerous surveys on the adherence to treatment guidelines in SA, usually focusing on specific medicines or health conditions, often only at the primary care level, which have found that treatment compliance amongst prescribers is poor [31, 37, 44–48]. However, the overall adherence to STGs/EML amongst nurses in a study by Sooruth et al. (2015) was found to be exceptionally high at 90.83% and all respondents self-reported having adhered to guidelines—it must also be noted that the study was conducted in 2013, before the hard copies of the STGs/EML were updated and were only made available electronically in late 2015 [35]. This study’s suboptimal compliance findings may further suggest that the phasing out of hardcopies at PHC level has not had the desired effect of increased accessibility to the latest guidelines.

Along with these findings, Sooruth et al. (2015) [36] also found that STGs/EML training amongst nurses in primary care was insufficient with less than 60% of professional nurses who had obtained a qualification that equipped them with knowledge in assessing, diagnosing, and treating patients in line with STGs/EML. Current in-service training programmes for professional nurses were found to be inadequate and ineffectively implemented as many nurses were employed without having attained the appropriate qualification and relied on inadequate “on-the-job” learning due to the increased demand for nurses and gross staff shortages at state facilities, leading to poor job satisfaction [49]. The availability of medicines has increased drastically, since the initial studies were conducted in 1998 in two South African provinces [50], as was evident upon auditing both PHC and hospital-level facilities.

### Limitations

This study was only conducted at public healthcare institutions and facilities within one of the many subdistricts of South Africa, thus may not paint a national picture nor provide a full evaluation of how the guidelines are utilized and implemented and may have implications when looking to generalize the results against wider populations across the country. Response to the self-administered questionnaire was the prerogative of the participant which could be subjective.

The prescription analysis relied on the record-keeping abilities of the prescribers which may have impacted the results—this was particularly seen in the lack of recording of diagnoses. As this study was done retrospectively, with access only to patient records, diagnoses could not be verified; and where the diagnosis was not recorded it was regarded as non-adherent to the guidelines which may have impacted the results as the treatment could either have been correctly or incorrectly prescribed. The study relied on quantitative data only thus did not fully investigate clinicians’ experience with the utilization of the STGs/EML. The prescribing of treatment is nuanced, and the various aspects of the patient should be considered, such as the severity of the condition, co-morbidities, concurrent medication, demographic data and the patient’s right to refuse treatment. These factors may have influenced the clinicians’ judgement to prescribe contrary to the treatment guidelines which was not considered as clinicians and patients were not interviewed.

### Strengths and novelty of study

There have been no studies conducted in SA to evaluate the implementation and utilization of the STGs/EML prior to the introduction of the STGs/EML policy and few thereafter. This study has shed light on aspects of the implementation outcomes of these policies at the service delivery level and provides a baseline by which future utilization and implementation studies can be compared to. The findings provide a grassroots picture of how the STGs/EML have been utilized and implemented at institutional (secondary and tertiary) and facility (primary) health care levels and may serve as a yardstick by which pertinent post-NHI policy outcomes can be measured.

### Recommendations

The healthcare landscape is constantly evolving with changing healthcare needs resulting in similarly changing learning needs of healthcare workers. To meet these needs, Norushe et al. (2004) [49] outlined interventions to improve in-service training programmes after conducting a study amongst PHC clinicians in East London, South Africa. Implementation of a skills assessment followed by an orientation programme for all new staff members on the use of the STGs and EML is recommended, as well as regular in-service training to reinforce and update the knowledge and skills of prescribers, particularly when the STGs/EML are updated, in line with interventions to monitor medicine policies recommended by the WHO [51], particularly at the PHC level. The reintroduction of hardcopies should be considered at PHC level to increase accessibility to the STGs/EML to ensure that the latest treatment guidelines are available until the accessibility to technology has improved. Access to mobile technology continues to grow [11], thus targeted training on how to access and utilize the EML Clinical Guide app should be prioritized for PHC prescribers to encourage usage of the mobile app.

The National Guidelines for the Establishment and Functioning of PTCs in South Africa was developed in 2019 to enact the National Policy for the Establishment and Functioning of PTCs of 2015. PTCs are responsible for ensuring that medicine related policies, such as the STGs/EML are implemented at all levels of care. A function of the PTC is to monitor and ensure rational medicine use and monitor prescribing practices in line with the STGs/EML [2], but the extent to which this is done at facility level has not been studied previously. It is essential that STGs/EML compliance and monitoring protocols are strengthened to ensure continued adherence to the guidelines, particularly regarding the compulsory recording of diagnoses in patient records. The utilization of electronic prescribing will improve adherence to guidelines, improve the quality of patient records and enhance the monitoring of prescriptions.

This study did not analyse the possible reasons for non-compliance to the STGs/EML, such as polypharmacy, incorrect dose and/or duration of treatment, lack of indication for prescribed medicine, incorrect treatment for diagnosis, etc.—targeting this area for future studies may identify gaps for targeted interventions and education. Future studies assessing the impact on health outcomes of patients upon introduction of the STGs/EML should be explored to provide a holistic picture of the impact that the utilization of STGs/EML has had. Research of this nature should be ongoing to act as a barometer to which successes and shortfalls of the implementation of such policies can be measured as this study can be replicated for a wider population to paint a national picture.

## Conclusion

The South African NDoH has been proactive in strengthening the healthcare system for its citizens as evidenced by the introduction of the STGs/EML, which has ensured good availability of essential medicines in the public sector and has been successful at providing adequate treatment for the prevalent health conditions in SA, thus has made essential medicines more accessible to the population, although, this study found that more robust monitoring and improved protocols are necessary to address the gaps found in the implementation of the STGs/EML to create more succinct and quality guidelines, and improve the utilization of the guidelines, thereby ensuring that quality and sustainable healthcare is accessible to all.

The STGs/EML provide evidence-based recommendations for rational prescribing of medicines, and while they cannot replace sound clinical judgement, they ensure standardized quality of patient care and promote access to quality and sustainable healthcare. As South Africa moves towards NHI implementation, utilization of the STGs/EML will guide treatment strategies and prevent excessive use of healthcare services to ensure the sustainability and affordability of the fund [4]. The research this study has provided intends to inform policies involved in the development and implementation of NHI and provide novel recommendations for improvement of the current Essential Medicines Programme as improved health outcomes are evidenced by accessible and quality medicines. Processes to monitor adherence to STGs/EML must be strengthened as these guidelines will dictate the healthcare services rendered upon NHI implementation.

## Data Availability

All data generated or analysed during this study are included in this article in tables.

## Abbreviations

AMD: Affordable Medicines Directorate
EML: Essential medicines list
NCS: National Core Standards
NDoH: National Department of Health
NDP: National Drug Policy
NHI: National Health Insurance
PHC: Primary Health Care
SA: South Africa
STGs: Standard treatment guidelines
UHC: Universal health coverage
WHO: World Health Organization
